# Evolution of Robustness to Noise and Mutation in Gene Expression Dynamics

**DOI:** 10.1371/journal.pone.0000434

**Published:** 2007-05-09

**Authors:** Kunihiko Kaneko

**Affiliations:** 1 Department of Pure and Applied Sciences, University of Tokyo, Tokyo Japan; 2 Complex Systems Biology Project, Exploratory Research for Advanced Technology (ERATO), Japan Science and Technology Agency (JST), Tokyo, Japan; University of East Piedmont, Italy

## Abstract

Phenotype of biological systems needs to be robust against mutation in order to sustain themselves between generations. On the other hand, phenotype of an individual also needs to be robust against fluctuations of both internal and external origins that are encountered during growth and development. Is there a relationship between these two types of robustness, one during a single generation and the other during evolution? Could stochasticity in gene expression have any relevance to the evolution of these types of robustness? Robustness can be defined by the sharpness of the distribution of phenotype; the variance of phenotype distribution due to genetic variation gives a measure of ‘genetic robustness’, while that of isogenic individuals gives a measure of ‘developmental robustness’. Through simulations of a simple stochastic gene expression network that undergoes mutation and selection, we show that in order for the network to acquire both types of robustness, the phenotypic variance induced by mutations must be smaller than that observed in an isogenic population. As the latter originates from noise in gene expression, this signifies that the genetic robustness evolves only when the noise strength in gene expression is larger than some threshold. In such a case, the two variances decrease throughout the evolutionary time course, indicating increase in robustness. The results reveal how noise that cells encounter during growth and development shapes networks' robustness to stochasticity in gene expression, which in turn shapes networks' robustness to mutation. The necessary condition for evolution of robustness, as well as the relationship between genetic and developmental robustness, is derived quantitatively through the variance of phenotypic fluctuations, which are directly measurable experimentally.

## Introduction

Robustness is ability to function against changes in the parameter of a system[1], [2], [3], [4], [5]. In a biological system, the changes have two distinct origins, genetic and epigenetic. The former concerns with genetic robustness, i.e., rigidity of phenotype against mutation, which is necessary to maintain a high fitness state. The latter concerns with fluctuation in number of molecules and external environment.

Indeed, phenotype of isogenic individual organisms is not necessarily identical. Chemotaxis[6], enzyme activities, and protein abundance[7], [8], [9], [10], [11] differ even among those sharing the same genotype. Recent studies on stochastic gene expression elucidated the sources of fluctuations [7]. The question most often asked is how some biological functions are robust to phenotypic noise[11], [12], while there may also be positive roles of fluctuations in cell differentiation, pattern formation, and adaptation[13], [14], [15], [16].

Noise, in general, can be an obstacle in tuning a system to the fittest state and maintaining it there. Phenotype of an organism is often reproducible even under fluctuating environment or under molecular fluctuations[2]. Therefore, phenotype that is concerned with fitness is expected to keep some robustness against such stochasticity in gene expression, i.e., robustness in ‘developmental’ dynamics to noise. Phenotype having a higher fitness is maintained under noise. How is such “developmental robustness” achieved through evolution? In the evolutionary context, on the other hand, another type of robustness, robustness to mutation need to be considered. When genetic changes occur, gene expression dynamics are perturbed so that phenotype with a high fitness may no longer be maintained. The “genetic robustness” concerns with the stability of a high-fitness state against mutation.

Whether these two types of robustness emerge under natural selection have long been debated in the context of developmental dynamics and evolution theory[3], [5], [17], [18], [19], [20], since the proposition of stabilization selection by Schmalhausen[21] and canalization by Waddington[22], [23], [24]. Are developmental robustness to noise and genetic robustness to mutation related? Is phenotypic noise relevant to attain robustness to mutation? In the present paper, we answer these questions quantitatively with the help of dynamical network model of gene expression.

Under the presence of noise in gene expression, phenotype as well as fitness, of isogenic organisms is distributed, usually following a bell-shaped probability function. When the phenotype is less robust to noise, this distribution is broader. Hence, the variance of this distribution, i.e., variance of isogenic phenotypic fluctuation denoted as *V_ip_*, gives an index for robustness to noise in developmental dynamics. On the other hand, robustness to mutation is measured from the fitness distribution over individuals with different genotypes. An index for it is given by variance of phenotypic fluctuation arising from diversity of genotypes in a population[25], [26], [27], denoted here as *V_g_*. This variance *V_g_* increases as the fraction of low-fitness mutants increases.

Here we show that evolution to increase both types of robustness is possible only when the inequality *V_ip_*≥*V_g_* is satisfied. Since the isogenic phenotypic fluctuation *V_ip_* increases with noise, this means that evolution of robustness is possible only when the amplitude of phenotypic noise is larger than some critical value as derived by *V_ip_*≥*V_g_*, implying a positive role of noise to evolution. We demonstrate that both the two variances *V_ip_* and *V_g_* decrease in the course of evolution, while keeping the proportionality between the two. This proportionality is consistent with an observation in a bacterial evolution experiment [16], [17], [18].

We explain the origin of the critical noise strength, by noting that smooth dynamical behavior free from a rugged potential landscape evolves as a result of phenotypic noise. When the noise amplitude is smaller than the threshold, we observe that low-fitness mutants are accumulated, so that robustness to mutation is not achieved. Generality and relevance of our results to biological evolution are briefly discussed.

### Theoretical Framework on Genetic-Phenotypic Relationship

In natural population, both the phenotype and genotype differ among individuals. Let us consider population distribution *P*(*x, a*) where *x* is a variable characterizing a phenotype and *a* is that for the corresponding genotype[18]. Here the phenotype *x* is responsible for the fitness of an individual, and the selection depending on *x* is considered as an evolutionary process. Since the phenotype differs even among isogenic individuals, the distribution *P*(*x*; *a* = *a*_0_) for a fixed genotype *a*_0_ has some variance. This isogenic phenotypic variance *V_ip_*, defined as the variance over clones, is written as 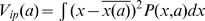, where 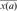 is the average phenotype of a clonal population sharing the genotype *a*, namely 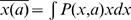. This variation of phenotype is a result of noise through the developmental process to shape the phenotype. If this variance is smaller, the phenotype is less influenced by noise, and thus *V_ip_* works as a measure of robustness of the phenotype against noise.

On the other hand, the standard evolutionary genetics [25], [26], [27] mainly studies the phenotypic variance due to genetic variation. It measures phenotypic variability due to diversity in genotypes in a population. This phenotypic variance by genetic variance, which is termed *V_g_* here, is then defined as the variance of the average 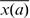, over genetically heterogeneous individuals. It is given by 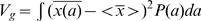, where *P*(*a*) is the distribution of the genotype *a* and <*x̅* > is the average of 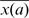 over genotypes *a*. While *V_ip_* is defined as variance over clones, i.e., individuals with the same genotype, *V_g_* comes from those with different genotypes. As *V_g_* is smaller, the phenotypic change by genetic variation is smaller. Hence *V_g_* gives a measure of robustness of the phenotype against mutation.

We have previously derived an inequality *V_ip_>V_g_* between the two variances, by assuming evolutionary stability of the population distribution *P*(*x, a*), that is preservation of single-peakedness through the course of evolution [18] (see Supporting Text S1). Indeed the single-peaked distribution collapses as *V_ip_* approaches *V_g_*, where the distribution is extended to very low values of *x* (fitness). In other words, error catastrophe occurs at *V_g_≈V_ip_*; (Here error catastrophe means accumulation of low-fitness mutants in the population after generations, and the term is used here by extending its original meaning by Eigen[28]). For each course of evolution under a fixed mutation rate, the proportionality between *V_g_* and *V_ip_* is derived, since the genetic variance increases roughly proportionally to the mutation rate[18].

Note, however, that the derivation of these relationships (*V_ip_*≥*V_g_*, error catastrophe at *V_g_≈V_ip_*, and proportionality between *V_g_* and *V_ip_* for a given course of evolution) is based on the existence of two-variable distribution function *P*(*x = phenotype, a = gene*), and the postulate that single-peaked distribution is maintained throughout evolution, which is not trivial. Hence the above relationships need to be examined by some models for evolution. In addition, *why does the population distribution extend to low-fitness values when the phenotypic fluctuation V_ip_ is smaller than V_g_*? Or, put it another way, why do systems with small phenotypic noise run into “error catastrophe”? In fact, the emergence of error catastrophe as a result of decreasing isogenic phenotypic fluctuation below *V_g_* may look rather counter-intuitive, since in general one expects fluctuation to perturb a system from the fittest state. The necessity of fluctuation for evolution to increase robustness to noise and to mutation needs theoretical examination.

## Methods

### Model

To study the proposed relationships, we need to consider seriously how the phenotype is shaped through complex “developmental process”. In the present paper, we use the term ‘development’, in a broad sense, including a process in uni-cellular organisms to reach cell division. It is a dynamical process to shape a phenotype at a ’matured’ state (where fitness is defined) from a given initial state. In general, this dynamic process is complex so that the process may not reach the identical phenotype due to the noise through this developmental process. This leads to the isogenic variance of the phenotype *V_ip_*. On the other hand, the equation governing the developmental process is varied as a result of mutation. The phenotype variance over a population with distributed genotypes gives *V_g_*.

We consider a simple model to satisfy the requirement on ‘development’ above. It consists of a complex dynamic process to reach a target phenotype under a noise which may alter the final phenotypic state. We do not choose a biologically realistic model that describes a specific developmental process, but instead take a model as simple as possible, to satisfy a minimal requirement for our study. Here we take a simplified model, borrowed from a gene regulatory network, where expression of a gene activates or inhibits expression of other genes under noise. These interactions between genes are determined by the network. The expression profile changes in time, and eventually reaches a stationary pattern. This gene expression pattern determines fitness. Selection occurs after introduction of mutation at each generation in the gene network. Among the mutated networks, we select a network with a higher fitness value. Since there is a noise term in the gene expression dynamics, fitness fluctuates even among the individuals with an identical gene network, which leads to the isogenic fluctuation *V_ip_*. On the other hand, the expression pattern varies by mutation in the network, and gives rise to variation in the average fitness, resulting in *V_g_*.

This simplified gene expression follows a typical switch-like dynamics with a sigmoid input-output behavior [29], [30], [31], [32], [33] widely applied in models of signal transduction[34] and neural networks[35] (For a related evolution model with discrete states, see e.g., [24]). The dynamics of a gene expression level *x_i_* is described by
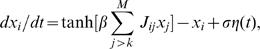
(1)where *J_ij_* = −1,1,0, and η(*t*) is Gaussian white noise given by <η(*t*)η(*t*′)> = *δ*(*t*−*t*′). *M* is the total number of genes, and *k* is the number of output genes that are responsible for fitness to be determined. The value of *σ* represents noise strength that determines stochasticity in gene expression (For simplicity we mainly consider the case that the noise amplitude is independent of *x_i_*, while inclusion of such *x*-dependence of noise amplitude does not alter the conclusion to be discussed). By following a sigmoid function *tanh*, *x_i_* has a tendency to approach either 1 or −1, which is regarded as “on” or “off” of gene expression. Even though *x* is defined over [−∞, ∞], it is attracted to the range [−1,1] (or slightly above or below the range due to the noise term). We consider a developmental process leading to a matured phenotype from a fixed initial state, which is given by (−1,−1,…,−1); i.e., all genes are off, unless noted otherwise. (This specific choice of initial condition is not important).

Let us define a fitness function so that gene expression levels (*x_i_*) for genes *i* = 1,2,…,*k*(<*M*) would reach an “on” state, i.e., *x_i_*>0. The fitness is maximum if all *k* genes are on after a transient time span *T_ini_*, and minimum if all are off. To be specific, we define the fitness function by
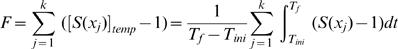
(2)where *S*(*x*) = 1 for *x*>0, and 0 otherwise, […]*_temp_* is time average between *t = T_ini_* and *t = T_f_* (The time average here is not important, because the gene expressions *x_i_* are fixed after some time, in most cases). Adoption of the value (*S*(*x_j_*)−1) after initial time *T_ini_* leads to the same result (. The fitness function takes the maximum value *F* = 0 when the selected pattern of gene expression (*x_i_*; *i* = 1,2,…,*k*) is always “on” and takes the minimum (*F = −k*) when all *k* genes are always off. Note that fitness is calculated only after time *T_ini_*, which is chosen sufficiently large so that the temporal average can be computed after the gene expression dynamics has fallen on an attractor. This initial time can be considered as the time required for developmental dynamics.

As the model contains a noise term, fitness fluctuates at each run, which leads to the distribution in *F*, even for the same network. Hence we obtain the distribution *p*(*F*; *g*), for a given network “g”, whose variance gives isogenic phenotypic fluctuation. At each generation, we compute the fitness *F* over *L* runs, to obtain the average fitness value *F̅* of a given network.

Now we consider the evolutionary process of the network. Since the network is governed by *J_ij_* which determines the ‘rule’ of the dynamics, it is natural to treat *J_ij_* as a measure of genotype. Individuals with different genotype have a different set of *J_ij_* At each generation there are *N* individuals with different sets of *J_ij_* For each individual network, we compute the average fitness *F̅*. Then we select the top *N_s_*(<*N*) networks that have higher fitness values. (The value *N/N_s_* corresponds to the selective pressure. As it is larger, the evolution speed increases. However, specific choice of this value itself is not important to the result to be discussed).

At each generation, mutation changes the network, i.e., changes *J_ij_* at a given mutation rate *µ*. We rewire the network at a given rate so that changes in *J_ij_* produce *N* new networks. (In most simulations, only a single path, i.e., a single pair of *i, j* is changed. The mutation rate can be lowered by changing a path only for some probability. Although it is important to the evolution speed and the error catastrophe point to be discussed, the conclusion to be discussed is not altered by specific choice of *µ*.)

Here we make *N/N_s_* mutants from each of the top *N_s_* networks, so that there will be *N* networks again for the next generation. From this population of networks we repeat the process of the developmental dynamics, fitness calculation, selection, and mutation (Instead of this simple genetic algorithm, we can also assume that the number of offspring increases with the fitness. This choice does not alter the conclusion to be presented).

Simulations start from a population of random networks with a given fraction of paths (for example, 50% of *J_ij_* are nonzero). At each generation, the *N* individuals have slightly different networks *J_ij_*, so that the values of *F̅* are different. We denote the fitness distribution over individuals with different genotype as *P*(*F̅* ). On the other hand, the fitness distribution for an identical network (“g”) is computed to obtain *p*(*F; g*).

### Remark:

Developmental dynamics and selection process in our model are too simple. Still, this model is relevant to examine general statement on phenotypic fluctuations, as the model at least captures complex dynamics giving rise to a phenotype, stochasticity in dynamics, mutation, and selection according to a given phenotype. Indeed, real gene expression dynamics depend on environmental conditions, and the fitness is defined as expression patterns to adapt each environmental condition. We have also carried out some simulations by imposing such fitness but the results to be discussed (with regards to *V_g_* and *V_ip_*) are invariant.

## Results

**Figure 1:**
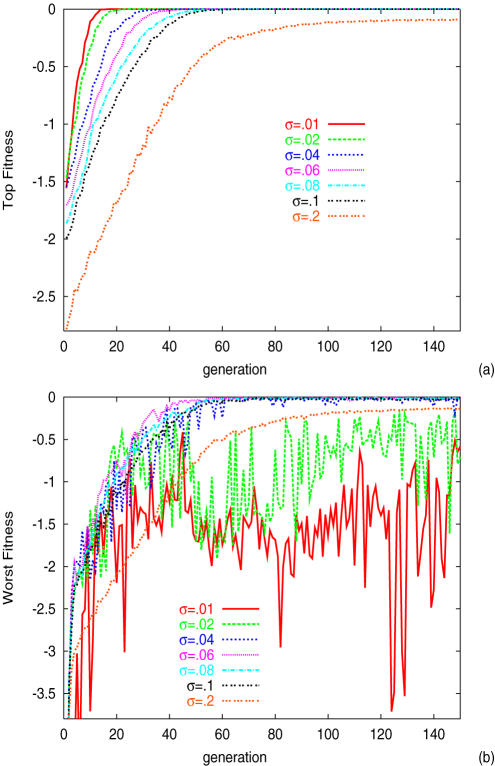
Evolutionary time course of the fitness *F̅*. The highest (a) and the lowest (b) values of the fitness *F̅* among all individuals that have different genotypes (i,e., networks *J_ij_*) at each generation are plotted. Plotted are for different values of noise strength, σ = 0.01,0.02,0.04,0.06,0.08,0.1, 0.2 with different color. Hereafter we mainly present the numerical results for *M* = 64 and *k* = 8. At each generation there are *N* individuals. *N_s_* = *N*/4 networks with higher values of *F̅* are selected for the next generation, from which mutants with a change in a single element *J_ij_* are generated. For the average of fitness, *L* runs are carried out for each. Unless otherwise mentioned, we choose *N* = *L* = 300, while the conclusion to be shown does not change as long as they are sufficiently large. (We have also carried out the selection process by *F* instead of *F̅*, but the conclusion is not altered if *N* is chosen to be sufficiently large.) Throughout the paper, we use *β* = 7.

**Figure 2:**
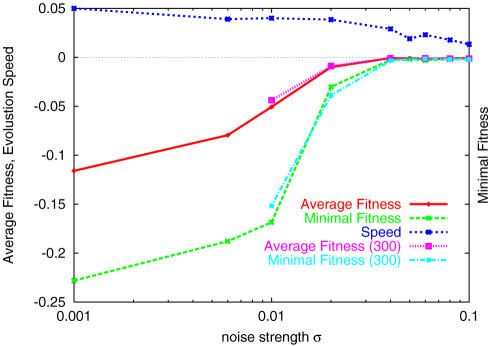
Average fitness <*F̅*>, lowest average fitness *F̅*_min_, evolution speed, and variance of the fitness *V_g_* are plotted against the noise strength *σ.* <*F̅*>, the average of the average fitness *F̅* over all individuals is computed for 100–200 generations (red cross, from the simulation with population of 100 individuals, and purple square from 300 individuals). The minimal fitness is computed from the time average of the least fit network present at each generation (green, from 100 population, and light blue from 300). The evolution speed is plotted, measured as the inverse of the time required for the top individual to reach the maximal fitness 0. *V_g_* is computed as the variance of the distribution *P*(*F̅*) at 200th generation.

**Figure 3:**
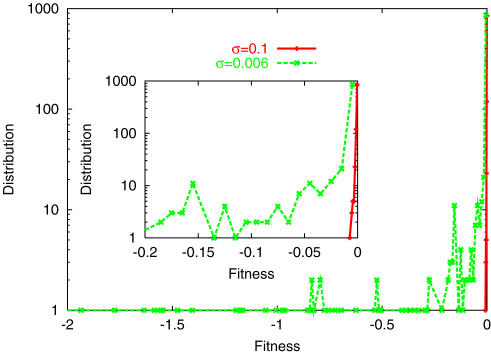
Distribution P(*F̅*) after 200 generations, for population of 1000 individuals. Inset is the magnification for −0.2<*F̅*<0. For high *σ* (red, with *σ* = 0.1), the distribution is concentrated at *F̅* = 0, while for low *σ* (green, with *σ* = 0.006), the distribution is extended to large negative values, even after a large number of generations.

Let us first see how the evolutionary process changes as a function of the noise strength *σ*. After generations, the peak position in *P*(*F̅*) increases, so that the top of *F̅* in the population approaches the highest value 0. Indeed, in all cases, the top group quickly evolves to the highest fitness state *F̅* = 0 (see Fig. 1a; even for *σ* = 0.2, the highest fittest value approaches 0 after a few hundred more generations.). The time necessary for the system to reach this state becomes shorter as the phenotypic noise decreases (see Fig. 2). On the other hand, the time evolution of the distribution function *P*(*F̅*) depends drastically on the noise strength *σ*. When *σ* is small, the distribution is broad and the existing individual with the lowest *F̅* remains at the low fitness state, while for large *σ*, even the individuals with the lowest fitness approach *F̅* = 0 (see Fig. 1b and Fig. 3). There is a threshold noise *σ_c_*(≈0.02), below which the distribution *P*(*F̅*) is broadened, as is discernible in the data of the variance of the distribution, *V_g_* in Fig. 2. Here, the top individuals reach the highest fitness, leaving others at the very low fitness state. As a result, the average fitness over all individuals, 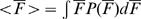 is low. <*F̅*> and the lowest fitness over individuals *F̅* *_min_*, after a sufficiently large number of generations, are plotted against *σ* in Fig. 2. The abrupt decrease in fitness suggests threshold noise *σ_c_*, below which low-fitness mutants always remain in the distribution. For *σ>σ_c_*, the distribution *P*(*F̅*) takes a sharp peak at *F̅* ∼0, where the variance is rather small. Distribution below and above *σ_c_* are displayed in Fig. 3. (This type of transition is also observed by increasing the mutation rate, while fixing the noise strength at *σ>σ_c_*).

Let us study the relationship between *V_g_* and *V_ip_* Here *V_ip_* is defined as variance from the distribution *p*(*F; genotype*), i.e., over individuals with the same genotype. As the distribution *p* depends on each individual with different genotype, the variance changes accordingly. Naturally, the top individual has a smaller variance, and the individual with lower fitness has a larger variance. As a measure of *V_ip_*, we used either the average of the variance over all individuals or the variance of phenotype from a gene network that is located closest to the peak in the distribution *P*(*F̅*). Both estimates of *V_ip_* do not differ much, and the following conclusion is drawn in both cases. *V_g_*, on the other hand, is simply the variance of the distribution *P*(*F̅*), i.e., over individuals having different genotypes present.

**Figure 4:**
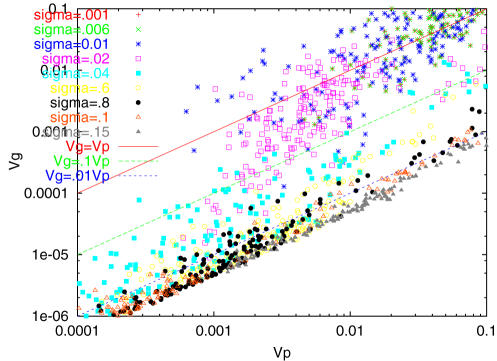
Relationship between *V_g_* and *V_ip_.* *V_g_* is computed from *P*(*F̅*) at each generation, and *V_ip_* by averaging the variance of *p*(*F; gene*) over all existing individuals. (We also checked by using the variance for such gene network that gives the peak fitness value in *P*(*F̅*), but the overall relationship is not altered). Plotted points are over 200 generations. For *σ*>*σ_c_*≈.02, both decrease with generations.

The relationship between *V_g_* and *V_ip_* thus evaluated is plotted in Fig. 4. We find that both the variances decrease through the evolutionary time course when *σ>σ_c_*, where we note:

(i) *V_ip_*>*V_g_* for *σ>σ_c_*.

(ii) *V_g_*∝*V_ip_* during the evolutionary time course under a fixed noise strength *σ*(>*σ_c_*) and a fixed mutation rate. As *σ* is lowered toward *σ_c_*, *V_g_* increases so that it approaches *V_ip_*.

(iii) *V_g_*≈*V_ip_* at *σ≈σ_c_*, where error catastrophe occurs.

In other words, the fittest networks maintaining a sharp distribution around the peak dominate only when *V_ip_*>*V_g_* is satisfied. As *σ* is decreased to *σ_c_*, *V_ip_* approaches *V_g_*, error catastrophe occurs and a considerable fraction of low-fitness mutants accumulates. Hence, the relationships proposed theoretically assuming evolutionary stability of a two-variable distribution function *P*(*x = phenotype, a = genotype*) is confirmed. Here, without introducing phenomenological assumptions, the three relationships are observed in a general stochastic gene-network model.

**Figure 5:**
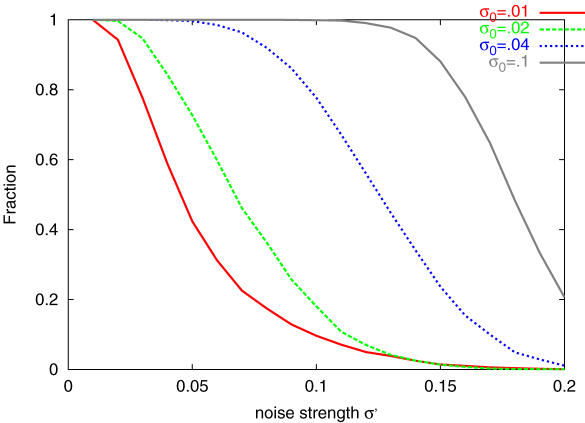
Dependence of the fraction of the runs that reach the target expression pattern. Networks that had the top fitness value under noise *σ* were simulated at a different noise *σ′.* We first generate a network as a result of evolution over 200 generations under the noise strength *σ*, and select such network *J_ij_* that has the top fitness value. Then we simulate this network under new noise strength *σ*′ from the initial condition −1,−1,…,−1 over 10000 runs, to check how many of them reach the target pattern (i.e., *x_i_*>0 for *i* = 1 to 8). Plotted is the fraction of such runs against the noise strength *σ*′. Different color corresponds to the value of the original noise strength *σ* used for the evolution of the network.

Why didn't the system maintain the highest fitness state under small phenotypic noise *σ<σ_c_*? To study the difference in dynamics evolved for different values of *σ*, we choose the top individual (network) that has evolved at *σ* = *σ*_0_, and place it under a different noise strength *σ* = *σ*′. In Fig. 5, we have plotted the fraction of runs giving rise to *F* = 0 under such circumstance. As shown, the successful fraction decreases when *σ*′ goes beyond *σ*_0_. In other words, the network evolved under a given noise strength successfully reaches the target gene expression up to that critical noise level, while it begins to fail doing so at a higher noise strength. Accordingly, the distribution *p*(*F; gene*) extends to lower values in fitness, when a network evolved under small phenotypic noise is developed under a higher noise level. On the other hand, the network evolved under high level noise maintains a high fitness value, even when the noise is lowered.

**Figure 6:**
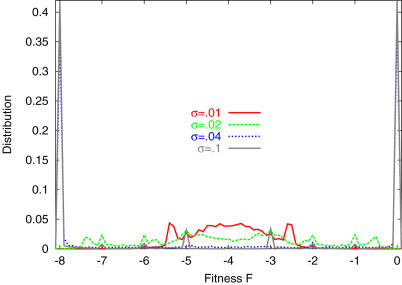
Distribution of the fitness value when the initial condition for *x_j_* is not fixed at −1, but is distributed over [−1,1] . We choose the evolved network as in Fig. 5, and for each network we take 10000 initial conditions, and simulated the dynamics (1) without noise to measure the fitness value *F* after the system reached an attractor (as the temporal average 400<*t*<500). The histogram is plotted with a bin size 0.1.

Next we study the basin structure of attractors in the present system. Note that an orbit of the network with the highest fitness, starting from the prescribed initial condition, is within the basin of attraction for an attractor corresponding to the target state (*x_i_*>0 for *i* = 1,…,*k*). Hence the basin of attraction for this target attractor is expected to be larger for the dynamics evolved under higher level noise. We have simulated the dynamics (1) for the evolved fittest network under zero noise, starting from a variety of initial conditions over the entire phase space, and measured the distribution of *F* at each attractor. The distribution is shown in Fig. 6 (Due to the symmetry against *x_j_* = 1↔*x_j_* = −1 in the model, the distribution is symmetric around *F* = −*k*/2 when all initial conditions are taken. In fact, by starting from *x_i_* = 1 for all *i*, the orbit reaches an attractor *x_j_*<0 for *j* = 1,…,*k*, resulting in *F* = −*k*). For the network evolved under *σ>σ_c_*, the distribution has a sharp peak at *F* = 0 (and *F* = −*k* due to the symmetry), with more than 40% attraction to each. On the other hand, for the networks evolved under *σ<σ_c_*, the peak height at *F*∼0 is very small, i.e., the basin for the attractor with *F* = 0 is tiny. There exist many small peaks corresponding to attractors with −*k*<*F*<0, having similar sizes of basin volumes. In fact, the basin volumes for attractors with −*k*<*F*<0 grow as *σ* is decreased, and are dominant for *σ<σ_c._*

### Dynamic Origin of Robust Evolution

**Figure 7:**
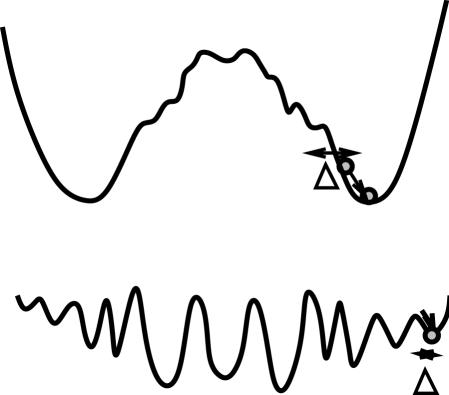
Schematic representation of the basin structure, represented as a process of climbing down a potential landscape. Δ is the magnitude of perturbation to jump over the barrier to a different attractor from the target. Smooth landscape is evolved under high level noise (above), and rugged landscape is evolved under low level noise (below).

The difference in the basin structure suggested by Fig. 6 is schematically displayed in Fig. 7. For the network evolved under *σ>σ_c_* there is a large, smooth attraction to the target state, while for the dynamics evolved under *σ<σ_c_*, the phase space is split into small basins. Let us consider the distance between the basin boundaries from a “target orbit” starting from −1,…,−1 and reaching *x_i_*>0 (for 1≤*i*≤*k*) which is defined by Δ here. The distance Δ remains small for the dynamics evolved under a low noise strength *σ<σ_c_*, and increases for those evolved under a higher noise. It is interesting to note that evolution influences the basin structure globally over the phase space, although the fitness condition is applied locally to an orbit starting from a specific initial condition.

**Figure 8:**
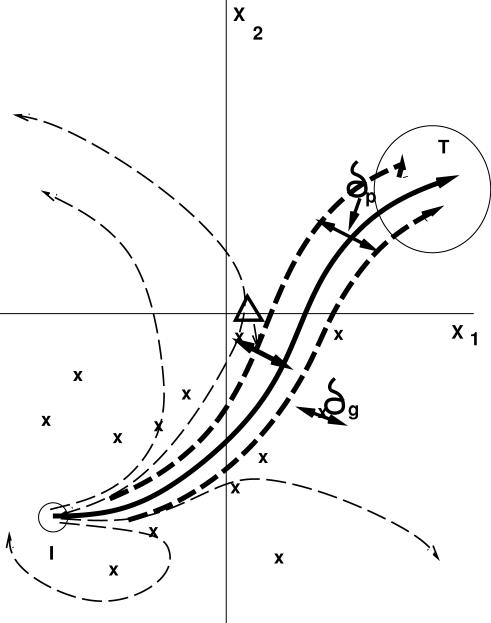
Schematic representation of an orbit in the phase space. The solid curve is an original orbit from the initial condition (I) to the target attractor (T). Dashed curves are orbits perturbed by noise. When orbits encounter turning points, they escape the original basin of attraction and may be caught in another attractor. Mutations, on the other hand, are able to move the position of turning points.

The results in Fig. 5 and Fig. 6 imply that the gene regulation networks that operate and evolve under noisy environment exhibit qualitatively different dynamics compared to those subjected to low level noise. In our model, the fitness of an individual changes when its gene expression *x_j_* for *j* = 1,…,*k* changes its sign. Recall that the fixed point solution *x_i_* = *tanh*(Σ*_j_J_ij_x_j_*) changes its sign when Σ*_j_J_ij_x_j_* in the sigmoid function changes its sign. This change may occur during the developmental dynamics by noise, and we call such points in the phase space ‘turning points’. When an orbit of eq.(1) passes over turning points, *x_j_* takes a negative value for some *j* (for 1≤*i*≤*k*) at the attractor (see Fig. 8 for schematic representation). Since there are many variables for gene expression and the values of *J_ij_* are distributed over −1 and 1, the term *tanh*(Σ*_j_J_ij_x_j_*) changes its sign at several points in the phase space {*x_j_*} generally. Hence there can be many turning points in the phase space. The fittest network with *F̅*≈0 chooses such orbits having no turning points within the noise range from the original orbit. An orbit from the fittest individual evolved under low-level noise encounters many turning points when subjected to noisy environment.

The average distance between the turning points and an orbit that has reached the target gene expression pattern is estimated by the distance Δ defined above. Recall that the distance Δ is small for the dynamics evolved under a low noise strength. Such dynamics, if perturbed by a higher level of noise, are easily caught in the turning points, which explains the behavior shown in Fig. 5.

Let us now discuss the relationship between *V_g_* and *V_ip_*. Noise disturbs an orbit so that it may go across the basin boundary (turning points) with some probability. We denote the standard deviation of the location of the orbit due to noise as *δ_p_*, which is proportional to the noise strength *σ*. Since the distance between the orbit and the basin boundary is deviated by *δ_p_*, and the fitness value drops when the orbit crosses the basin boundary, the variance *V_ip_* is estimated to be proportional to (*δ_p_*/Δ)^2^.

Next, we discuss how the mutation in the network influences the dynamics. When the network is altered, i.e., a path is added or removed as a result of mutation in *J_ij_*, there exists a variation of the order of 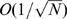 in the threshold function term in eq.(1). This leads to a deviation of the location of turning points (or basin boundary). We denote this deviation as *δ_g_*, which increases with the mutation rate. The variance *V_g_* is estimated to be proportional to (*δ_g_*/Δ)^2^, with the same proportion coefficient as that between *V_ip_* and (*δ_g_*/Δ)^2^.

Under the presence of noise, there is a selective pressure to avoid the turning points (basin boundaries) that exist within the distance *δ_p_* from the “target” orbit. This leads to an increase in Δ. However, if *δ_p_* is larger than *δ_p_*, the memory of this distance between the target and the boundaries will not be propagated to the next generation, due to large perturbation to the original network by the mutation. Hence increase in Δ (i.e., increase in robustness) is expected only if *δ_p_>δ_g_*. Since *δ_p_* and *δ_g_* increase with the noise strength *σ* and the mutation rate *µ* respectively, there exists a critical noise strength *σ_c_* beyond which this inequality is satisfied. From the relationship between *δ_p,g_* and *V_ip,g_*, the condition for robust evolution is rewritten as *V_ip_>V_g_*.

When the condition *V_ip_>V_g_* (i.e., *σ>σ_c_*) is satisfied, the system increases Δ during evolution. We have computed the temporal evolution of basin distribution. With generations, the distribution evolves from the pattern at a low level noise in Fig. 7, to that at large *σ* characterized by enhanced peak at *F* = 0. Accordingly Δ increases with generations. Recall that *V_ip_*∼(*δ_p_*/Δ)^2^, and *V_g_*∼(*δ_g_*/Δ)^2^, both variances decrease with generations, while *V_ip_/V_g_* is kept constant.

## Discussion

We have demonstrated the inequality and proportionality between *V_g_* and *V_ip_*, through numerical evolution experiment of a gene network. As phenotypic noise is decreased and the inequality *V_ip_>V_g_* is broken, low-fitness mutants are no longer eliminated. This is because the mutants fail to reach the target gene expression pattern, by crossing the boundary of the basin of attraction to the target. When the amplitude of the noise is larger, on the other hand, the networks of the dynamics with a large basin volume for the target attractor are selected and thus mutants with lower fitness are removed successively through the selection process. Hence noise increases developmental robustness through evolution, together with genetic robustness.

Although we used a specific example to demonstrate the relationship between *V_ip_* and *V_g_* and the error catastrophe, we expect this relationship to be generally applicable to systems satisfying the following conditions:

(i) Fitness is determined through developmental dynamics.

(ii) Developmental dynamics is sufficiently complex so that its orbit, when deviated by noise, may fail to reach the state with the highest fitness.

(iii) There is effective equivalence between mutation and noise in the developmental dynamics with regards to phenotype change.

Note that the present system as well as the previous cell model[18] satisfies these conditions. The condition (i) is straightforward in our model, and the condition (ii) is satisfied because of the complex dynamics having many turning points in the phase space. Noise in developmental dynamics sometimes perturbs an orbit to cross the basin boundary so as to escape from attraction to the target gene expression pattern, while a mutation in the network may also induce such failure, by shifting basin boundaries. Hence the condition (iii) is satisfied.

When developmental process fails due to phenotypic noise, the fitness function takes a low value. Evolution under noise acts to prevent such failure within the range of noise. On the other hand, due to the condition (iii), mutation may also lead to such lethality. When the effect of mutation goes beyond the range given by the phenotypic noise, mutants with very low fitness values begin to accumulate. Hence there appears a threshold level of phenotypic noise below which low-fitness (or deleterious) mutants accumulate (or threshold mutation rate beyond which such mutants accumulate). In this sense, we expect that for robust evolution, the inequality *V_g_<V_ip_* must be satisfied in order for the low-fitness mutants to be eliminated. Violation of the inequality leads to accumulation of low-fitness (or deleterious) mutants, a phenomenon known as error catastrophe[28]. Only under the presence of noise in the developmental process, systems acquire robustness through evolution. In other words, developmental robustness to stochasticity in gene expression implies genetic robustness to mutation. Quantitative analyses of stochasticity in protein abundance during the laboratory evolution of bacteria are possible [17], [36]. By carefully measuring the variation *V_g_* of given phenotype in mutants, and comparing it with that of isogenic bacteria, *V_ip_*, one can examine the validity of our conclusion between *V_g_* and *V_ip_*.

It is worthwhile to mention that in a class of theoretical models, fitness landscape is given as an explicit continuous function of a gene sequence (e.g., energy function in a spin glass[37]), where a minute change in sequence does not lead to a drastic change in fitness. On the other hand, in a system satisfying (i) and (ii), a small change in genotype (e.g., a single change in the network path) may result in a large drop in fitness, since fitness is determined after the developmental dynamics. Indeed, there may appear mutants with very low fitness values from an individual with a high fitness value, only by a single change of a path in the network. Such deleterious mutations are also observed in nature[27].

It is interesting to note that a larger basin of attraction to a target attractor (with the highest fitness value) is formed through a mutation and selection process. As a result, dynamics over the entire phase space are simplified to those having only a few attractors, even though the fitness function is given locally without scanning over the entire phase space. When the time-course is represented as a motion along a potential surface, our results suggest that the potential landscape becomes smoother and simpler through evolution, and loses ruggedness after generations. Indeed, existence of such global attraction in an actual gene network has recently been reported in yeast cell-cycle[38].

Such smooth landscape was also studied in protein folding[39], [40]. Saito et al.[41] observed an evolutionary process from a rugged to the so-called funnel-like landscape in an interacting spin system abstracting protein folding dynamics. Under a general framework of statistical mechanics[42], a relationship between the degree of variance in coupling coefficients *J_ij_* between spins (corresponding to *V_g_*) and the temperature (i.e., phenotypic noise for spin *x_i_*, corresponding to *V_ip_*) is formulated. Such relationship may be relevant to understand the relationships between *V_g_* and *V_ip_* in our study.

According to established Fisher's theorem on natural selection, evolution speed of phenotype is proportional to the phenotypic variance by genetic variation, *V_g_* [25], [26], [27]. The demonstrated proportionality between *V_ip_* and *V_g_* then suggests that the evolution speed is proportional to the isogenic phenotypic fluctuation, as is also supported by an experiment on bacterial evolution in a laboratory[17] and confirmed by simulations of a reaction network model of a growing cell[18].

Isogenic phenotypic fluctuation is related to phenotypic plasticity, which is a degree of phenotype change in a different environment. Positive roles of phenotypic plasticity in evolution have been discussed[20], [43], [44], [45]. Since susceptibility to the environmental change and the phenotypic fluctuation are positively correlated according to the fluctuation-response relationship[16,46.47], our present results on the relationship between phenotypic fluctuations and evolution imply, inevitably, a relationship between phenotypic plasticity and evolution akin to genetic assimilation proposed by Waddington[22].

## Supporting Information

Text S1 Derivation of General Relationship on Fluctuations. Mathematical derivation on general relationships on phenotypic variances is presented.
(0.05 MB PDF)
