# Supplementary material for: Evolution of Robustness to Noise and Mutation in Gene Expression Dynamics
Source: PLoS One. 2007 May 9;2(5):e434. doi: 10.1371/journal.pone.0000434 (PMC1855988; doi:10.1371/journal.pone.0000434)
Supplement: Text S1 — Derivation of General Relationship on Fluctuations. Mathematical derivation on general relationships on phenotypic variances is presented. (0.05 MB PDF) [file pone.0000434.s001.pdf]

Supporting Information:

Here we briefly review the relationship between isogenic phenotypic variance V_{ip} and genetically induced variation V_g. (see also [16,18]).

We consider the distribution both in phenotype and genotype, which is represented as a parameter a here. Through the evolutionary process, the dominant genotype a changes, and the dominant phenotype $x_0(a)$ changes accordingly. Now, to investigate evolution both with regards to the distribution of phenotype and genotype, we introduce a two-variable distribution $P(x, a)$. In this analysis, the assumption is made that there is a two-variable distribution in genotype and phenotype.

Now, V_{ip} is the variance of x, which can be written as $V_{ip}(a) = \int (x - \overline{x(a)})^2 P(x,a)dx$, where $\overline{x(a)}$ is the average phenotype of a clonal population sharing the genotype a, namely $\overline{x(a)} = \int P(x,a)xdx$. V_g is then defined as the variance of the average $\overline{x(a)}$, over genetically heterogeneous individuals, given by $V_g = \int (\overline{x(a)} - <\overline{x}>)^2 P(a)da$, where $P(a)$ is the distribution of genotype a, and $<\overline{x}>$ as the average of $\overline{x(a)}$ over genotypes.

Assuming the Gaussian distribution, we consider the distribution $P(x; a)$ as follows:

$$P(x,a) = N \exp[-\frac{(x - X_0)^2}{2\alpha(a)} + C(a - a_0)(x - X_0) - \frac{1}{2\mu}(a - a_0)^2], \tag{1}$$

where N is a normalization constant. Here the Gaussian distribution $\exp(-\frac{1}{2\mu}(a - a_0)^2)$ represents the distribution of genotype around $a = a_0$. The variance here is (in a suitable unit) nothing but the mutation rate μ. The above equation can then be rewritten as

$$P(x,a) = N \exp[-\frac{(x - X_0 - C(a - a_0)\alpha)^2}{2\alpha(a)} + (\frac{\alpha C^2}{2} - \frac{1}{2\mu})(a - a_0)^2]. \tag{2}$$

The second assumption we make is that at each stage of the evolutionary process, the distribution has a single peak in (x, a) space. In order for this distribution to have a single peak (i.e., not to be flattened along the direction of a) the following condition (besides $\alpha > 0$) should be satisfied:

$\frac{\alpha(a_0)C^2}{2} - \frac{1}{2\mu} \leq 0$, i.e.,

$$\mu \leq \frac{1}{C^2\alpha(a_0)} \equiv \mu_c \tag{3}$$

This means that the mutation rate has an upper bound beyond which the distribution does not have a peak in the genotype-phenotype space.

Now we investigate the phenotypic variance due to the genotype distribution. First, we consider the average $\overline{x}_a$ over the distribution $P(x, a)$ for a given fixed a, and then we consider the distributions of $\overline{x}_a$ for changes in a around a_0. The variance V_g of this distribution of $\overline{x}_a$ is given by

$$V_g = <(\overline{x}_a - \overline{x}_{a_0})^2> . \tag{4}$$

Then noting that

$$\overline{x}_a \equiv \int x \exp(-V(x,a))dx = X_0 + C(a - a_0), \tag{5}$$

we obtain

$$V_g = <(\overline{x}_a - \overline{x}_{a_0})^2> = C^2 <(\delta a)^2> = C^2 \mu. \tag{6}$$

Accordingly, the inequality $\mu < 1/(C^2\alpha(a_0))$ is rewritten as

$$V_g \leq \alpha(a_0). \tag{7}$$

Recall that α is the phenotypic variance $<(\delta x)^2>$ of isogenic individuals, V_{ip}. Then the above inequality is written as

$$V_g \leq V_{ip}. \tag{8}$$

For a more general derivation of the above inequality, see [18].

Since the genetic variance in the population $<(\delta a)^2>$ is proportional to the mutation rate, the above inequality sets an upper bound for mutation rate, beyond which the single peak distribution breaks down. Let us denote this threshold mutation rate as μ_{max}. Recall $V_g \propto \mu$, and we get

$$V_g = \frac{\mu}{\mu_{max}} V_{ip}. \tag{9}$$

Thus we get the proportionality between V_g and V_{ip} through a given course of evolution with a fixed mutation rate.

Fisher's theorem[25-27] states that the evolution speed is proportional to the phenotypic variance caused by genetic variation, V_g. Following the above expression, one can conclude that the evolution speed is proportional to μV_{ip}, which is nothing but the prediction by evolutionary fluctuation-response relationship[16-18].
